# The identification of a novel frameshift insertion mutation in the *EXT1* gene in a Chinese family with hereditary multiple exostoses

**DOI:** 10.1002/ccr3.6298

**Published:** 2022-09-08

**Authors:** Wanlu Liu, Xinwei Shi, Yuqi Li, Fuyuan Qiao, Yuanyuan Wu

**Affiliations:** ^1^ Department of Obstetrics and Gynecology Tongji Hospital, Tongji Medical College, Huazhong University of Science and Technology Wuhan China

**Keywords:** *EXT1*, frameshift insertion mutation, hereditary multiple exostoses

## Abstract

To identify the pathogenic gene variation in a Chinese family with Hereditary Multiple Exostoses (HME). By examining blood‐sourced DNA and clinical manifestations of the proband and his family members, the whole exome sequencing (WES) and Sanger sequencing were used to detect possibly pathogenic mutations. A novel heterozygous mutation (c.325dup) was identified in exon 1 of the *exostosin 1 (EXT1)* gene from the proband and the affected family members. And we found this mutation was absent in all the unaffected family members. This c.325dup mutation is in the exon 1 domain of the *EXT1* gene and the change of p.C109Lfs*80 cause the early termination of protein translation. The identification of the novel frameshift insertion mutation (c.325dup) expands the mutation spectrum of HME, which provides new evidence for HME diagnosis.

## Graphical abstract

Hereditary multiple exostoses (HME) is a rare skeletal disorder characterized by a widespread distribution of osteochondromas originating from the metaphyses of long bones. The proband was diagnosed with HME by clinical manifestations and gene test.

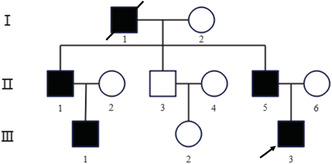

## 1 INTRODUCTION

Hereditary Multiple Exostoses (HME) is a rare orphan autosomal‐dominant pediatric disorder with a prevalence of about 1:50000.^1^, ^2^ The disease is estimated to occur more frequently in males (male‐to‐female ratio, 1.5:1).^3^ HME is characterized by the formation of osteochondromas (nonmalignant cartilage‐capped bony tumors) or exostoses within the perichondrium next to the growth plates of long bones, ribs, hip, and vertebrae in very young and adolescent patients.^4^ Osteochondromas can turn into chondrosarcomas or osteosarcomas that can be life‐threatening in about 2% of the patients.^5^, ^6^ The current clinical treatment of HME is commonly to resect the symptomatic chondrosarcomas or osteochondromas and to ameliorate the associated skeletal defects. The etiological treatment is not yet available as the evidence on etiological diagnosis of HME is limited.

The identification of likely pathogenic genes associated with HME was reported in the past. Wu et al.^7^ reported that the majority of the studied patients carried mutations in the exostosin‐1 (*EXT1*) and exostosin‐2 (*EXT2*) genes.^8^ *EXT1* (OMIM: 608177) consists of 11 exons and spans about 312 kb at 8q24,^9^ while *EXT2* (OMIM: 608210) comprises 16 exons and is located at 11p11.2, spanning about 150 kb.^10^ It is known that the genes belonging to the *EXT* multigene family are ubiquitously expressed and act as tumor suppressors. The proteins encoded by *EXT* family genes are involved in the adhesion and/or polymerization of heparin sulfate (HS) chains at HS proteoglycans (HSPGs).^11^, ^12^, ^13^ To date, over 650 mutations in *EXT1* and *EXT2* have been reported, most of which are nonsense, frameshift, or splice site, resulting in the synthesis of truncated EXT proteins with no suppression activity.^14^, ^15^

In this study, we identified a novel frameshift insertion mutation in the proband, which is absent both in ClinVar or Human Genome database and current clinical reports. The novel frameshift insertion mutation found in the proband was also confirmed in the affected individuals but not in the unaffected individuals. Further, the PolyPhen and SIFT analysis were used to evaluate the effect of the frameshift insertion mutation, with a support result of obtaining a dysfunctional protein from the mutated gene. Therefore, we concluded that the genetic variation caused by the frameshift insertion mutation could be associated with the pathogenicity of HME in this pedigree. The finding could be used as a new support for prenatal diagnosis for preventing the birth defect incidence of HME.

## 2 MATERIALS AND METHODS

### 2.1 Clinical report

**FIGURE 1:**
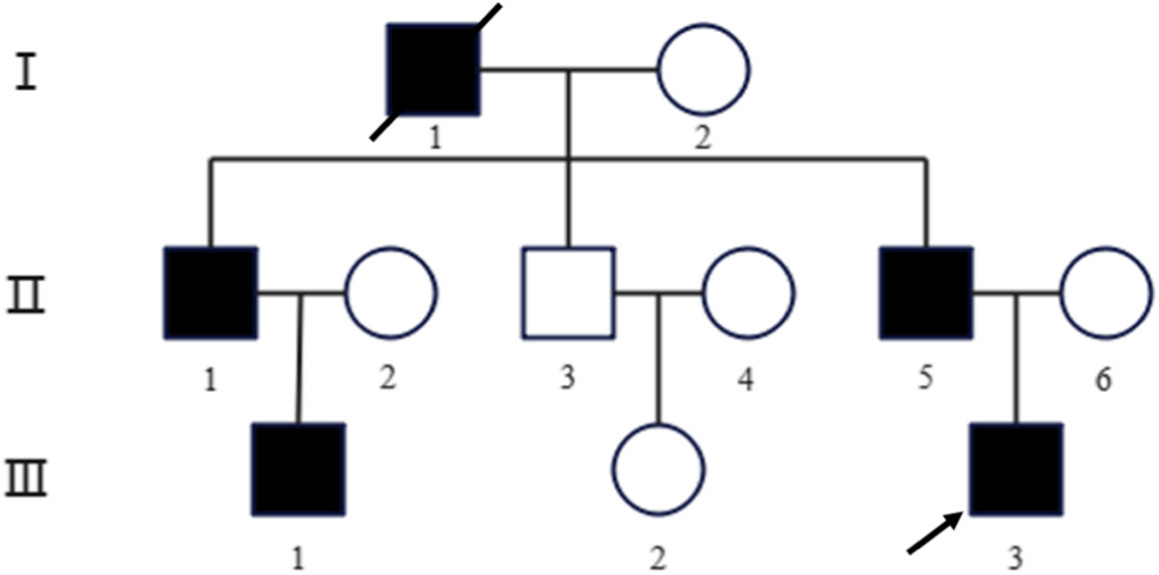
Pedigree of a family with Hereditary multiple exostoses (HME). Empty symbols indicate unaffected individuals, filled symbols indicate affected individuals, and oblique lines indicate deceased. The black arrow points the proband (III‐3).

The proband (III‐3) was a 21‐year‐old male who was admitted to Tongji Hospital of Tongji Medical College of Huazhong University of Science and Technology (Wuhan, China) due to his leg deformity with limited physical activity. The clinical examination concluded that the proband was diagnosed with HME at orthopedics department. Three generations of the patient family were included in this study with five individuals having HME (Figure 1). We have done a telephone follow‐up and learned that grandfather found many masses around the knee joint in adolescence, and then the bones were deformed and unable to move normally. The affected individuals (the grandfather, two uncles, proband, and elder male cousin) had found many masses around the different joints by X‐ray. The clinical manifestations of the affected individuals were pain and joint deformity, and movement disorder, some of whom received surgical treatment.

### 2.2 DNA extraction

Five ml of whole blood sample were collected from the proband (III‐3) and the other family members (I‐2, II‐1, II‐2, II‐3, II‐4, II‐5, II‐6, III‐1, and III‐2) individually using EDTA‐anticoagulant vacuum blood collection tubes. Genomic DNA was extracted from peripheral blood leukocytes of the collected blood samples using a DNA extraction kit (TianGen) according to the manufacturer's instructions.

### 2.3 Mutation screening and familial validation

The genomic DNA (50 ng) of each sample was enzyme‐digested into around 200 bp of fragments. The DNA fragments were end‐repaired (the 3′ end was added to one adenine base) and were ligated with barcoded sequencing adaptors. And the ligated fragments of about 320 bp were captured by XP beads. After polymerase chain reaction (PCR) amplification, the DNA fragments were hybridized by Nano whole exome sequencing (WES) according to the manufacturer's Protocol. The hybridized DNA products were eluted and then subjected to PCR amplification and purification as a DNA library for each sample. Next, the libraries were quantified by real‐time quantitative PCR (qPCR), and size distribution was determined using Nano WES (Berry Genomics). Finally, NovaSeq 6000 platform (Illumina, with 150 bp pair‐end sequencing mode) was used for genomic DNA sequencing. Raw image files were processed using CASAVA v1.82 for base calling and generating raw data. The sequencing reads were aligned to the human reference genome (hg19/GRCh37) using Burrows–Wheeler Aligner tool and PCR duplicates were removed using Picard v1.57 (http://picard.sourceforge.net/). Verita Trekker® Variants Detection System by Berry Genomics and the third‐party software GATK (https://software.broadinstitute.org/gatk/) was employed for variant calling. Variant annotation and interpretation were conducted by ANNOVAR^16^ and the Enliven® Variants Annotation Interpretation System (a comprehensive tool called Sprinkle was developed and authorized by Berry Genomics) was used for Copy Number Variations (CNV) calling. It includes XHMM PCA method (sequencing noise removal), CNV Kit fix module (GC and bias correction), and copy number calculation. The identification of CNV was performed in exons and long segment areas.

Mutation Surveyor Demo software version 4.0 was used to analyze the sample sequences comparing with the reference sequences from the National Center for Biotechnology Information (NCBI) (*EXT1*: NM_000127.2; *EXT2*: NM_000401). The detected variants were further evaluated by the PolyPhen and SIFT software to determine their associations with the pathogenicity of HME. The genetic variants were further examined for parents by Sanger sequencing when the pathogenic gene variations were detected in the proband.

## 3 RESULTS

### 3.1 Genetic variations associated with HME

**FIGURE 2:**
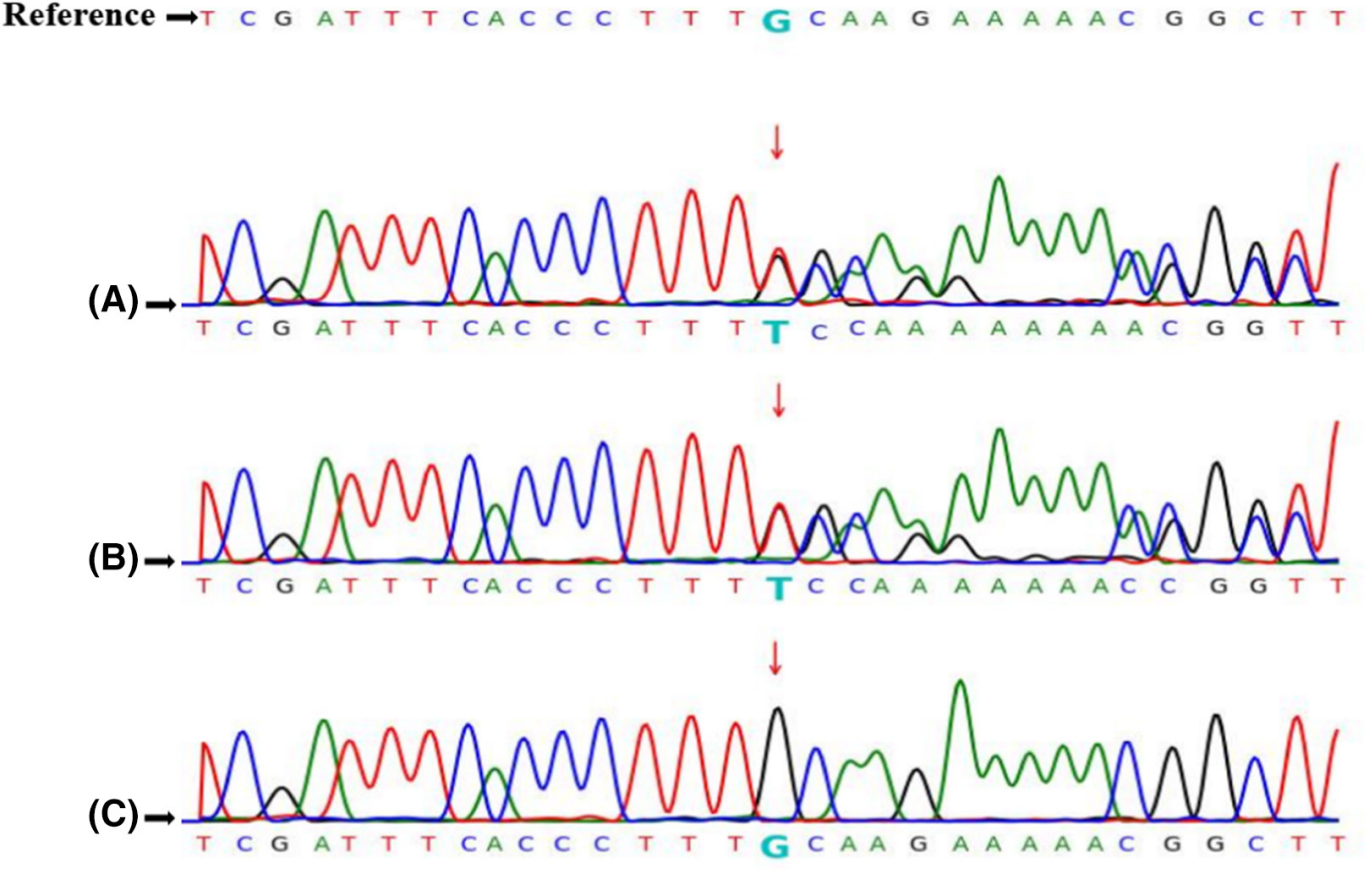
Identification of a novel mutation (c.325dup) in the EXT1 gene. (A) The novel frameshift insertion mutation was identified in the EXT1 gene of the proband (III‐3). (B) The novel frameshift insertion mutation in the affected family members (II‐1, II‐5, III‐1). (C) Absence of the novel frameshift insertion mutation in the unaffected family members (I‐2, II‐2, II‐3, II‐4, II‐6, III‐2).

The exons of the *EXT1, EXT2,* and the other 59 genes associated with HME (recommended by American College of Medical Genetics and Genomics, ACMG SF v2.0, shown in Table 1)^17^ were screened in the proband (III‐3) to reveal the possible pathogenic gene variants of HME. No mutations were discovered in the *EXT2* and the other 59 genes. However, a heterozygous frameshift insertion mutation in exon 1 of the *EXT1* gene was detected (c.325dup, duplication of 325 T), which was predicted to cause the early termination of protein translation (p.C109Lfs*80, premature codon stopping) (Figure 2A). We also screened the HME‐associated genes for the other family members, including affected individuals (II‐1, II‐5, and III‐1) and unaffected individuals (I‐2, II‐2, II‐3, II‐4, II‐6, and III‐2). The heterozygous frameshift insertion mutation of the proband was also detected in the affected family members but not in the unaffected family members (Figure 2B,C).

**TABLE 1:** 59 genes except *EXT* associated with HME were listed in ACMG SF v2.0

*ACTA2*	*ACTC1*	*APC*	*APOB*	*ATP7B*	*BMPR1A*	*BRCA1*	*BRCA2*
*CACNA1S*	*COL3A1*	*DSC2*	*DSG2*	*DSP*	*FBN1*	*GLA*	*KCNH2*
*KCNQ1*	*LDLR*	*LMNA*	*MEN1*	*MLH1*	*MSH2*	*MSH6*	*MUTYH*
*MYBPC3*	*MYH11*	*MYH7*	*MYL2*	*MYL3*	*NF2*	*OTC*	*PCSK9*
*PKP2*	*PMS2*	*PRKAG2*	*PTEN*	*RB1*	*RET*	*RYR1*	*RYR2*
*SCN5A*	*SDHAF2*	*SDHB*	*SDHC*	*SDHD*	*SMAD3*	*SMAD4*	*STK11*
*TGFBR1*	*TGFBR2*	*TMEM43*	*TNNI3*	*TNNT2*	*TP53*	*TPM1*	*TSC1*
*TSC2*	*VHL*	*WT1*					

We examined the reported gene variants of *EXT1* gene in Human Gene Mutation Database (HGMD) (shown in Table 2). The heterozygous frameshift insertion mutation identified in our study was absent in HGMD, thus it is recognized as a new gene variant associated with HME.

**TABLE 2:** A summary of different *EXT1* gene variants present in the publicly available version of HGMD (Professional Release 2021.12)

Mutation type	Number of Mutations (publicly available via http://www.hgmd.cf.ac.uk/ac/index.php)
Frameshift	262 (44.3%)
Nonsense	128 (21.6%)
Missense	67 (11.3%)
Canonical‐splice	58 (9.8%)
Gross deletions/insertions/duplications (>20 bp)	52 (8.8%)
Noncoding	10 (1.7%)
In‐frame	9 (1.5%)
Initiation	2 (0.3%)
Splice	2 (0.3%)
Regulatory	1 (0.2%)
Synonymous	1 (0.2%)
Total of variants	592

### 3.2 Prediction of protein function

**FIGURE 3:**
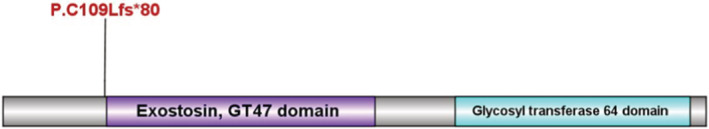
A schematic diagram of the functional domain of the EXT1 protein

The PolyPhen and SIFT analysis showed that the c.325dup frameshift insertion mutation in *EXT1* gene was predicted to cause the early termination of protein translation p.C109Lfs*80. The protein of *EXT1*, is encoded by 746 amino acids, of which two functional domains according to the InterPro website query (https://www.ebi.ac.uk/interpro/). Based on this result, a schematic diagram of the functional domain of the protein is made as Figure 3.

### 3.3 Genetic counseling

The unaffected family member (III‐2) requested a premarital genetic counseling in the study to determine her HME‐risk status. Since the genetic mode of HME is autosomal‐dominant inheritance, we suggested that her offspring could be less likely to get the HME‐associated gene variant from the mother according to the Mendelian law when she marries a healthy male. Differently, when the proband (III‐3) and the elder male cousin (III‐1) marry a healthy female, the offspring of them suffering from this disease accounts for 50%.

## 4 DISCUSSION

Genetic investigations suggested that gene variations in the *EXT1* and *EXT2* genes were often associated with HME, being responsible for 70%–95% of the cases. Mutations in *EXT1* account for 56%–78% of HME cases, whereas mutations in *EXT2* are detected in 21%–44% of cases.^14^ The *EXT1* gene mutation carriers tend to show more severe symptoms of HME and a greater risk for malignant transformation than the *EXT2* gene mutation carriers did.^18^, ^19^ The Human Gene Mutation Database, (HGMD, http://www.hgmd.org) stored the *EXT1* gene variants associated with HME that were published in the peer‐reviewed literature. To date, there are 592 gene mutation sites in *EXT1* gene presented in (HGMD), which were categorized into 11 categories of mutation type as shown in Table 2. In this study, we identified a novel frameshift insertion mutation of exon 1 of the *EXT1* gene in a Chinese pedigree with HME. This gene variant was not present in ClinVar/HGMD and current clinical reports.

Our result suggested that the novel frameshift insertion mutation c.325dup of the *EXT1* gene could cause the early termination of protein translation (p.C109Lfs*80). The protein of *EXT1*, is encoded by 746 amino acids, of which two functional domains according to the interpro website query (https://www.ebi.ac.uk/interpro/). The first is exostin, GT47 domain, located in amino acid 111–395 region. The second is glycosyl transferase 64 domain, located in amino acid 480–729 region. The change of the protein in this paper starts with leucine at position 109, and the translation is terminated after the 80th position, which has a great impact on the function and structure of the protein. Strong pathogenicity evidence (PVS1) shows that this mutation could change gene open reading frame, resulting in the loss of protein function. Moderate pathogenicity evidence (PM2) of this mutation was undetectable in Shenzhou genome database, Exome Aggregation Consortium (ExAC), 1000 Genomes Project (1000GP) and HGMD. The pathogenic evidence (PP4) of the identified mutation was consistent with the phenotype of HME. Generally, the combined evidences (PVS1 + PM2 + PP4) supported that this novel mutation was pathogenic^20^, ^21^, ^22^ but the origin of this pathogenic mutation is unknown.

In WES analysis on other family members, including affected individuals (II‐1, II‐5, and III‐1) and unaffected individuals (I‐2, II‐2, II‐3, II‐4, II‐6, and III‐2), we found that the identified mutation of the proband (III‐3) was present in all the affected family members but not present in the unaffected family members. We assumed that the pathogenic mutation of the affected individuals (III‐1 and III‐3) was obtained from their fathers (II‐1, II‐5), and the mutation of their fathers was inherited from the grandfather (I‐1) since the genetic type of HME is autosomal‐dominant inheritance. Based on the current evidence, the offspring of III‐1 and III‐3 will carry this novel mutation with HME incidence of 50% regardless of gender, while the offspring of the unaffected member (III‐2) will not carry the HME‐risk mutation.

Current methods of HME treatment are based on surgical removal of exostoses, especially those symptomatic or causing damage and irritation to the local structures. In the case of asymptomatic osteochondromas, no therapy is implemented. Surgical treatment intends to relieve chronic pain reported by most patients and prevent them from skeletal deformities, which often include growth asymmetry, resulting in limb length discrepancy. Moreover, it is performed to restore the motion of joints, improve circulation hampered by vessel compression, or for cosmetic purposes.^23^, ^24^ Other potential and promising treatment targets include selective agonist of retinoic acid receptor γ and Hedgehog signaling pathways or an enzyme heparinase according to the mutation of the gene, out of which any can turn out to be a potential treatment target.^25^, ^26^, ^27^ In this paper, only the proband and his elder male cousin have been performed surgical treatment because of the leg deformity with limited physical activity.

## 5 CONCLUSION

In sum, we identified a novel *EXT1* frameshift insertion mutation c.325dup on exon 1 and confirmed that the mutation could have pathogenic effect on gene expression level. Our finding enriches the HME mutation spectrum and provides scientific supports for premarital and prenatal diagnosis in the future.

## AUTHOR CONTRIBUTIONS

Wanlu Liu involved in conceptualization, resources, data curation, wrote the original draft. Xinwei Shi involved in methodology and software. Yuqi Li involved in formal analysis and visualization. Fuyuan Qiao involved in project administration. Yuanyuan Wu involved in supervision, funding acquisition, review, and editing.

## FUNDING INFORMATION

This study was supported by The National Key Research and Development Program of China (2018YFC1002904). This project was named as “Research and development of new technologies for intrauterine diagnosis and treatment of major fetal diseases”.

## CONFLICT OF INTEREST

The authors declare that they have no conflict of interest.

## ETHICAL APPROVAL

The study was approved by the Research Ethics Committee of Tongji Hospital (TJ‐C20201121) affiliated to Tongji Medical College of Huazhong University of Science and Technology. Informed consents were obtained from all the participants.

## CONSENT

The patient and his family provided the written informed consent before the study began.

## Data Availability

The datasets generated and/or analyzed during the current study are available from the corresponding author on reasonable request.

## References

[1] Wicklund CL , Pauli RM , Johnson DR , et al. Natural history of hereditary multiple exostoses. Am J Med Genet. 1995;55(1):43‐46. doi:10.1002/ajmg.1320550113 7702095

[2] Schmale GA , Conrad EU , Raskind WH . The natural history of hereditary multiple exostoses. J Bone Joint Surg Am. 1994;76(7):986‐992. doi:10.2106/00004623-199407000-00005 8027127

[3] D'Arienzo A , Andreani L , Sacchetti F , Colangeli S , Capanna R . Hereditary multiple exostoses: current insights. Orthop Res Rev. 2019;11(12):199‐211. doi:10.2147/ORR.S183979 31853203 PMC6916679

[4] Ryckx A , Somers JF , Allaert L . Hereditary multiple exostosis. Acta Orthop Belg. 2013;79(6):597‐607. 24563962

[5] Porter DE , Simpson AHRW . The neoplastic pathogenesis of solitary and multiple osteochondromas. J Pathol. 1999;188(2):119‐125. doi:10.1002/(SICI)1096-9896 10398153

[6] Porter DE , Lonie L , Fraser M , et al. Severity of disease and risk in malignant change in hereditary multiple exostoses. J Bone Joint Surg Br. 2004;86(7):1041‐1046. doi:10.1302/0301-620x.86b7.14815 15446535

[7] Wu YQ , Heutink P , de Vries BB , et al. Assignment of a second locus for multiple exostoses to the pericentromeric region of chromosome 11. Hum Mol Genet. 1994;3(1):167‐171. doi:10.1093/hmg/3.1.167 8162019

[8] Wuyts W , Van Hul W , Wauters J , et al. Positional cloning of a gene involved in hereditary multiple exostoses. Hum Mol Genet. 1996;5(10):1547‐1557. doi:10.1093/hmg/5.10.1547 8894688

[9] Ludecke HJ , Ahn J , Lin X , et al. Genomic organization and promoter structure of the human EXT1 gene. Genomics. 1997;40(2):351‐354. doi:10.1006/geno.1996.4577 9119404

[10] Clines GA , Ashley JA , Shah S , Lovett M . The structure of the human multiple exostoses 2 gene and characterization of homologs in mouse and Caenorhabditis elegans. Genome Res. 1997;7(4):359‐367. doi:10.1101/gr.7.4.359 9110175 PMC139145

[11] Lind T , Tufaro F , McCormick C , Lindahl U , Lidholt K . The putative tumor suppressors EXT1 and EXT2 are glycosyltransferases required for the biosynthesis of heparan sulfate. J Biol Chem. 1998;273(41):26265‐26268. doi:10.1074/jbc.273.41.26265 9756849

[12] McCormick C , Leduc Y , Martindale D , et al. The putative tumour suppressor EXT1 alters the expression of cell‐surface heparan sulfate. Nat Genet. 1998;19(2):158‐161. doi:10.1038/514 9620772

[13] Busse M , Feta A , Presto J , et al. Contribution of EXT1, EXT2, and EXTL3 to heparan sulfate chain elongation. J Biol Chem. 2007;282(45):32802‐32810. doi:10.1074/jbc.M703560200 17761672

[14] Jennes I , Pedrini E , Zuntini M , et al. Multiple osteochondromas: mutation update and description of the multiple osteochondromas mutation database (MOdb). Hum Mutat. 2009;30(12):1620‐1627. doi:10.1002/humu.21123 19810120

[15] Ciavarella M , Coco M , Baorda F , et al. 20 novel point mutations and one large deletion in EXT1 and EXT2 genes: report of diagnostic screening in a large Italian cohort of patients affected by hereditary multiple exostosis. Gene. 2013;515(2):339‐348. doi:10.1016/j.gene.2012.11.055 23262345

[16] Wang Y , Su P , Hu B , et al. Characterization of 26 deletion CNVs reveals the frequent occurrence of micro‐mutations within the breakpoint‐flanking regions and frequent repair of double‐strand breaks by templated insertions derived from remote genomic regions. Hum Genet. 2015;134(6):589‐603. doi:10.1007/s00439-015-1539-4 25792359

[17] Kalia SS , Adelman K , Bale SJ , et al. Recommendations for reporting of secondary findings in clinical exome and genome sequencing, 2016 update (ACMG SF v2.0): a policy statement of the American College of Medical Genetics and Genomics. Genet Med. 2017;19(2):249‐255. doi:10.1038/gim.2016.190 27854360

[18] Francannet C , Cohen‐Tanugi A , Merrer L , et al. Genotype‐phenotype correlation in hereditary multiple exostoses. J Med Genet. 2001;38(7):430‐434. doi:10.1136/jmg.38.7.430 11432960 PMC1757186

[19] Alvarez CM , De Vera MA , Heslip TR , et al. Evaluation of the anatomic burden of patients with hereditary multiple exostoses. Clin Orthop Relat Res. 2007;462(9):73‐79. doi:10.1097/BLO.0b013e3181334b51 17589361

[20] Abou Tayoun AN , Pesaran T , DiStefano MT , et al. Recommendations for interpreting the loss of function PVS1 ACMG/AMP variant criterion. Hum Mutat. 2018;39(11):1517‐1524. doi:10.1002/humu.23626 30192042 PMC6185798

[21] Biesecker LG , Harrison SM . The ACMG/AMP reputable source criteria for the interpretation of sequence variants. Genet Med. 2018;20(12):1687‐1688. doi:10.1038/gim.2018.42 29543229 PMC6709533

[22] Rajarshi G , Steven MH , Heidi LR , et al. Updated recommendation for the benign stand‐alone ACMG/AMP criterion. Hum Mutat. 2018;39(11):1525‐1530. doi:10.1002/humu.23642 30311383 PMC6188666

[23] Stieber JR , Dormans JP . Manifestations of hereditary multiple exostoses. J Am Acad Orthopaedic Surgeons. 2005;13(2):110‐120. doi:10.5435/00124635-200503000-00004 15850368

[24] Bovée JVMG , Hogendoorn PCW , Wunder JS , Alman BA . Cartilage Tumours and bone development: molecular pathology and possible therapeutic targets. Nat Rev Cancer. 2010;10(7):481‐488. doi:10.1038/nrc2869 20535132

[25] Herget GW , Kontny U , Saueressig U , et al. Osteochondrom und multiple osteochondrome. Radiologe. 2013;53(12):1125‐1136. doi:10.1007/s00117-013-2571-9 24129968

[26] Heuzé Y , Holmes G , Peter I , Richtsmeier JT , Jabs EW . Closing the gap: genetic and genomic continuum from syndromic to nonsyndromic craniosynostoses. Curr Genet Med Rep. 2014;2(3):135‐145. doi:10.1007/s40142-014-0042-x 26146596 PMC4489147

[27] Pacifici M . Hereditary multiple exostoses: new insights into pathogenesis, clinical complications, and potential treatments. Curr Osteoporos Rep. 2017;15(3):142‐152. doi:10.1007/s11914-017-0355-2 28466453 PMC5510481

